# A novel defined m7G regulator signature to investigate the association between molecular characterization and clinical significance in lung adenocarcinoma

**DOI:** 10.3389/fonc.2022.897323

**Published:** 2022-08-02

**Authors:** Yi Dong, Yingge Li, Yi Yao, Qibin Song

**Affiliations:** Cancer Center, Renmin Hospital of Wuhan University, Wuhan, China

**Keywords:** m7G, lung adenocarcinoma, prognosis, immunity, mutation

## Abstract

**Background:**

About170 chemical modifications to RNAs have been identified, which significantly affect gene expression. Dysregulation of RNA modifications induced by abnormal expression or mutations in RNA modifiers might result in cancer. The most frequent RNA modifications are N6-methyladenosine (m6A), 5-methylcytosine (m5C), and N7-methylguanosine (m7G). Lung cancer is the leading cause of cancer-related deaths globally. The present study aimed to investigate whether the expression of the m7G-related genes is linked to lung cancer cases with lung adenocarcinoma (LUAD), which accounts for about 40% of lung cancer cases.

**Methods:**

A total of 12 m7G-related differentially expressed genes (DEGs) were identified in LUAD patients by The Cancer Genome Atlas (TCGA). The least absolute shrinkage and selection operator (LASSO) Cox regression method was used to build a four-gene risk model. Then, LUAD patients in the TCGA cohort were divided into low- and high-risk groups based on their risk scores for subsequent molecular and clinical research.

**Results:**

Compared to the low-risk group, the high-risk group had a decreased overall survival (OS) (P=0.047). The risk score and stage were independent factors for predicting the OS of LUAD (P=0.0004 and P<0.0001, respectively). Gene ontology and Kyoto Encyclopedia of Genes and Genomes analyses based on the two groups showed that the DEGs were metabolically and hormonally related. The high-risk group showed a higher mutation rate and lesser immune cell infiltration, especially in TP53, KRAS, and MET. The expression level of PD-L1 and CTLA4 was high in the high-risk group (P<0.05). The high-risk group is more sensitive to anti-cancer therapy with lower IC50 and higher immunophenoscore (IPS).

**Conclusions:**

In this study, we developed a novel LUAD stratification model based on m7G-related genes that successfully predicts the prognosis of LUAD patients and serves as a guide for clinically personalized treatment.

## Introduction

Lung cancer is one of the most common types of cancer. As a leading cause of cancer mortality worldwide, several investigations have been conducted to manage the disease, including early diagnosis, advanced instruments, and improved treatments (1). Lung cancer is a heterogeneous tumor classified into different histological subtypes, including adenocarcinoma, squamous carcinoma (commonly referred to as non-small cell lung cancer), and small cell lung cancer. Comprehensive biological research has improved our understanding of this disease and contributed to the development of medications, such as targeted therapy and immunotherapy, ushering in a new era of precision medicine (2). Despite significant advances, several issues, from the mechanism to effective therapies, need to be resolved. In addition to oncogene activation, epigenetic factors, such as DNA methylation, chromatin architecture, histone modifications, and noncoding RNA regulation, play a role in lung cancer development (3). Most eukaryotic cells go through a range of biological processes known as co-transcriptional or post-transcriptional modifications. A recent study indicated that mRNA translation modulation plays a critical role in cancer progression (4). In tRNA, >90 distinct modified nucleosides have been identified; N7-methylguanosine (m7G) is one of the most conserved molecules (5). Protein synthesis is regulated by tRNA modification, essential for correct codon identification and reading frame preservation. Moreover, dysregulated tRNA modification has been linked to mitochondrial illnesses, neurological disorders, and cancer (6). Sustaining proliferative signaling, evading growth suppressors, resisting cell death, enabling replicative immortality, inducing angiogenesis, activating invasion and metastasis, reprogramming energy metabolism, and evading immune destruction are among the eight hallmarks of cancer in the multistep development of human tumors (7). Furthermore, some studies have shown that tRNA modification dysregulation may have an impact on all these processes. For example, the overexpression of tRNA alters the tRNA expression landscape and boosts cellular metabolic activity and proliferation rates *in vitro* (8). The whole-exome sequencing technology has provided a wealth of knowledge about genes and diseases, and another study found that tRNA^Glu^UUC and tRNA^Arg^CCG were elevated in the metastatic breast cancer cell lines, suggesting that it could boost the translational efficiency of disease-promoting genes, leading to a pro-metastatic state (9). A recent next-generation sequencing study discovered a group of tRNAs that can distinguish between normal and breast cancer samples as well as favorable prognosis from poor prognosis, implying them as putative cancer prognostic indicators (10). Some studies demonstrated that m7G promotes the translation of specific cell cycle regulatory and carcinogenic mRNAs enriched in the corresponding m7G-tRNA cognate codons, preventing ribosome pausing and ribosome collision-mediated translation inhibition (11). The RNA methyltransferase complex METTL1/WDR4 (methyltransferase like 1; ortholog of Trm8/WD repeat domain 4) catalyzes the m7G modification of a subset of tRNAs that are upregulated in certain malignancies (12). The levels of METTL1/WDR4 and m7G tRNA modifications are increased in human intrahepatic cholangiocarcinomas (ICCs), and cell cycle promoting mRNAs, such as those encoding *cyclin A2*, *cyclin D2*, *CDK6*, *CDK8*, and oncogenic mRNAs such as epidermal growth factor receptor (*EGFR*), were most translationally affected by m7G tRNAs (13). Currently, several studies are underway to uncover new fascinating cancer functioning secrets. However, the specific mechanism underlying lung cancer is yet to be elucidated. Herein, we conducted a comprehensive investigation to compare the expression levels of these m7G-related genes in normal and lung adenocarcinoma (LUAD) samples, to further analyze the prognostic significance and interaction between m7G and tumor microenvironment (TME), and to provide directions for future research.

## Materials and methods

### Dataset collection and procession

**Figure 1:**
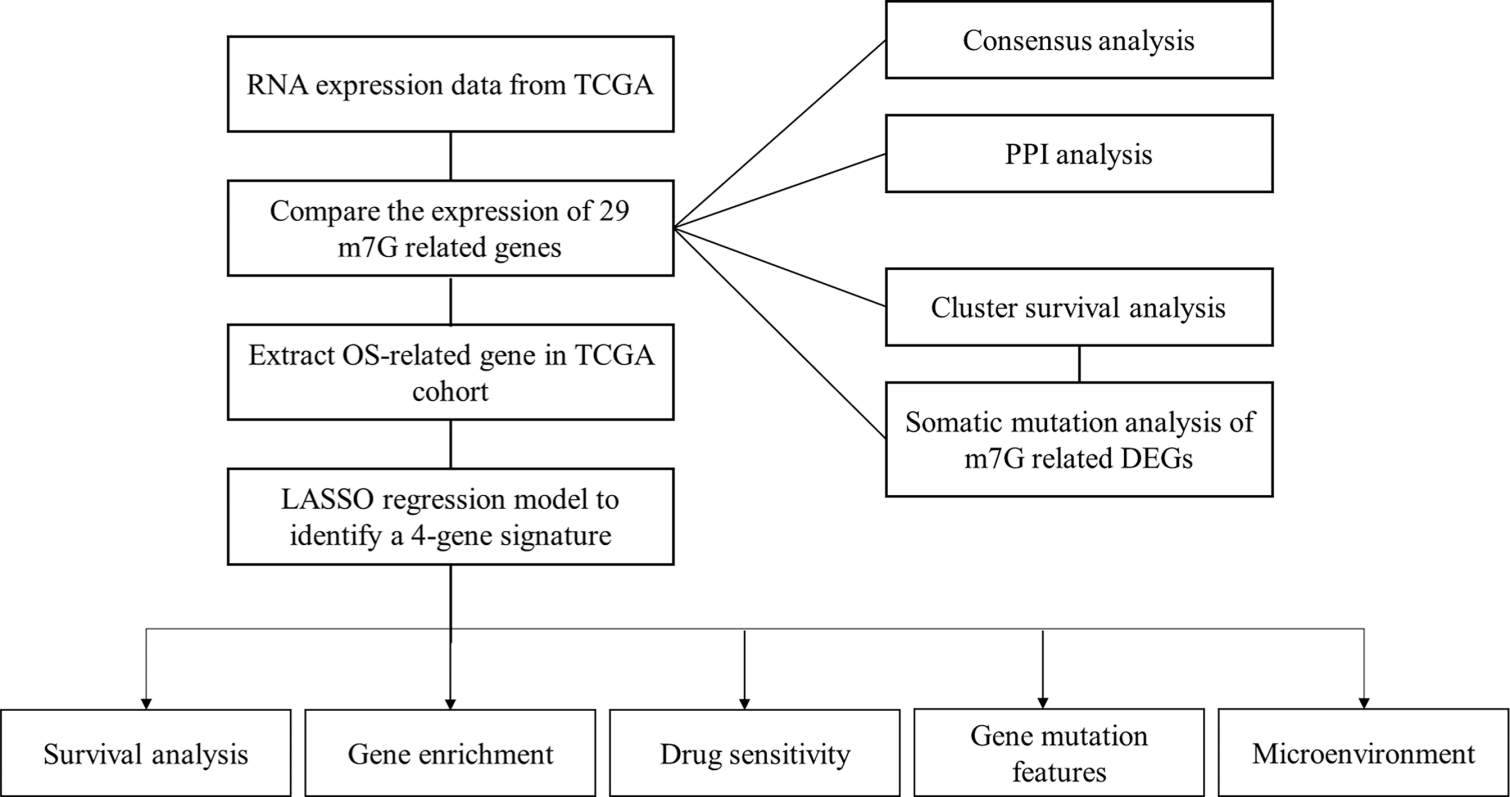
Workflow diagram. Specific workflow of data analysis.

The m7G regulators were collected from previously published studies (Supplemental Files m7G gene) and the GSEA website (http://www.gsea-msigdb.org/). The dataset of RNA sequencing (RNA-seq) data and corresponding clinical features of patients were obtained from the TCGA databases (https://portal.gdc.cancer.gov/repository). The workflow is shown in **Figure 1**.

### Identification of m7G-related regulators with differential expression

Herein, we retrieved 29 m7G-related genes from The Cancer Genome Atlas (TCGA) dataset. Differentially expressed genes (DEGs) with |log_2_FC| > 0.5 and false discovery rate (FDR)< 0.05 were identified using the “limma” program. The expression of all these m7G-related genes is shown in the heatmap. The Search Tool for the Retrieval of Interacting Genes (STRING) (https://string-db.org/) was used to create protein-protein interaction (PPI) networks for the m7G-related genes, which were then visualized by Cytoscape. In order to determine the central elements, we identified the top five hub genes from the PPI network *via* the MCC technique in the Cytohubba plugin.

### Development of the m7G-related gene prognostic model

Unsupervised consensus clustering was performed to cluster the tumor samples into subgroups based on the expression matrix of m7G regulators using the ConsensusClusterPlus R package to identify the m7G regulator-mediated subtypes. Clustering was performed using the following parameters: number of repetitions = 50; pItem = 0.8 (resampling 80% of any sample); pFeature = 1 (resampling 80% of any protein); clustering algorithm = k means method. We created a heatmap of differentially expressed m7G-associated genes and clinical characteristics based on this clustering method. To narrow down the putative genes and build a predictive model, researchers used the least absolute shrinkage and selection operator (LASSO) Cox regression model (R package “glmnet”). Subsequently, the m7G-related DEGs and their coefficients were retained, and the penalty parameter (λ) was determined using the minimum criteria. The risk score was calculated after centralization and standardization (applying the “scale” function in R) of TCGA expression data, and the risk score formula was as follows: Risk Score = $\sum_{i}^{n}X_{i}\times Y_{i}$ : X: coefficients, Y: gene expression level). Next, we employed Cox regression analysis to evaluate the correlation between each gene and survival status in the TCGA cohort to assess the prognostic value of the DEGs. To prevent omissions and for further studies, we set the P-value at 0.2. Thus, genes with P-values< 0.05 were extracted for survival analysis using the online tool (http://kmplot.com/analysis/), and we calculated an immunologic infiltration score for these genes in LUAD. The data were obtained from UCSC (https://xenabrowser.net/). The R package of “psych” (version 2.1.6) was used to calculate the immunological score for each oncogene.

### Independent prognostic analysis of the model

The TCGA LUAD patients were classified into low- and high-risk subgroups based on the median risk score, and the overall survival (OS) was compared between the two subgroups using Kaplan-Meier analysis. The “prcomp” function in the “stats” R package was used for principal component analysis (PCA) based on the risk model-associated gene signature. A 1-, 2-, and 3-year receiver operator characteristic (ROC) curve study was conducted using the “survival” and “timeROC” R packages. Univariate and multivariable Cox regression models were used to analyze the risk score and clinical parameters, such as age and stage.

### Functional enrichment analysis of DEGs based on the model

According to the median risk score, LUAD patients in the TCGA cohort were divided into two categories. Selective criteria (|log_2_FC| ≥ 1 and FDR< 0.05) were used to identify the DEGs between the subgroups derived from the risk model. The “clusterProfiler” software was used to conduct the GO enrichment analysis, and the web tool Enrichr was used to conduct Kyoto Encyclopedia of Genes and Genomes (KEGG) enrichment analysis based on these DEGs (https://maayanlab.cloud/Enrichr/).

### Estimation of TME and mutation between the subgroups

The tumor mutation burden (TMB) score for each patient was generated using the somatic mutation data of LUAD patients collected from the TCGA database. The TMB was compared between the two groups, and the survival probability was combined with the risk level. The Estimation of Stromal and Immunological Cells in Malignant Tumors using Expression Data (ESTIMATE, https://bioinformatics.mdanderson.org/estimate/index.html) platform was used to compute the stromal, immune, and ESTIMATE scores of samples in the TCGA database, which were validated in multiple ways. To further examine the mechanism of immunotherapy, we compared the expression of immune-checkpoint-related genes, including *PD-L1* and *CTLA4*, and evaluated the tumor immune dysfunction and exclusion (TIDE) score to identify the patients who would benefit from immune checkpoint inhibitor (ICI). The TIDE score was acquired after uploading the gene expression file as the instruction, and the immunophenoscore was computed *via* The Cancer Immunome Atlas (https://tcia.at/) (14). To determine the proportion of invading immune cells and analyze the efficiency of immune-related pathways, single-sample Gene Set Enrichment Analysis (ssGSEA) was carried out using the “gsva” software package. Furthermore, the immune cell proportion score for each group was compared to predict the efficacy of immunotherapy. The drug sensitivity was evaluated using the “pRRophetic” R package and the concentration that inhibited 50% of cellular growth (IC50).

### Statistical analysis

For DEG analysis, the “limma” R package was utilized, and the Pearson’s chi-square test was employed to evaluate the differences in the composition. Next, we employed the Kaplan-Meier (K-M) method with a two-sided log-rank test to compare the patient OS between subgroups. To assess the risk model’s independent predictive efficiency, we used univariate and multivariate Cox regression models. The immune cell infiltration and immunological pathway activation were assessed using the Mann-Whitney test. All statistical studies were carried out using the R programming language (v4.1.2).

## Results

### Identification of DEGs between normal and tumor tissues

**Figure 2:**
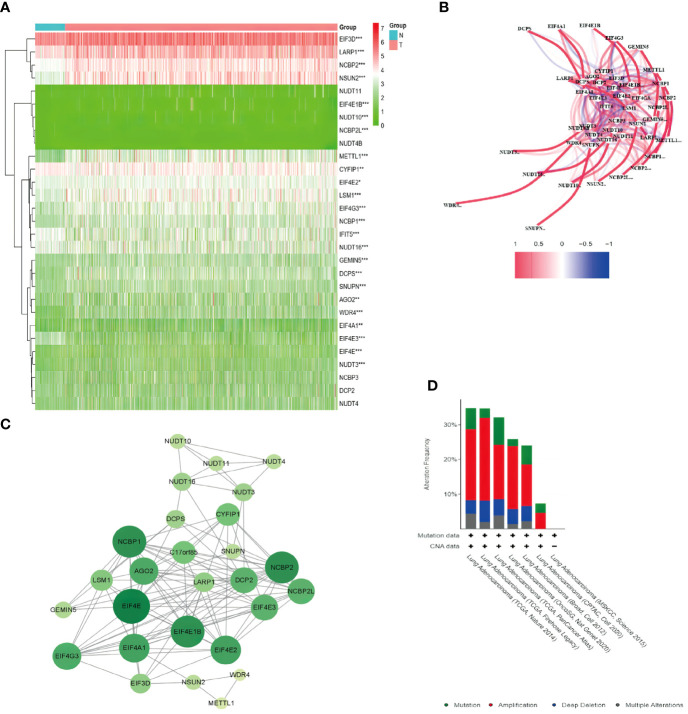
Expression of the 29 m7G-related genes and the interactions among the genes. **(A)** Heatmap of the m7G-related genes between the normal (N, brilliant blue) and the tumor tissues (T, red). P-values are shown as *P < 0.05, **P < 0.01; ***P < 0.001; green represented low expression, while red represented high expression. **(B)** Correlation network of the m7G-related genes (red line: positive correlation; blue line: negative correlation. The intensity of the colors reflected the strength of the relevance). **(C)** PPI network showed the interactions of the m7G-related genes (the bigger and deeper the circle is, the most important gene it might be). **(D)** Comparison of mutation data among different datasets *via* cBioPortal.

The expression data of 29 m7G-related genes in 59 normal and 535 LUAD tissues were extracted from the TCGA database, and 12 DEGs that met the criteria (|log_2_FC| > 0.5 and FDR< 0.05) were identified: *DCPS*, *EIF4E1B*, *EIF4E3*, *EIF4G3*, *LARP1*, *LSM1*, *METTL1*, *NCBP1*, *NCBP2*, *NCBP2L*, *NSUN2*, and *WDR4*. Among these, *EIF4E3* was downregulated, and all the others were upregulated in tumor specimens. The RNA expression of these genes was presented as heatmaps in **Figure 2A**. To further explore the interactions of these m7G-related regulators, we conducted a PPI analysis. A total of 28 nodes and 118 edges were detected in the network when the minimum required interaction score for the PPI analysis was set at 0.4 (**Figure 2B**). In **Figure 2C**, the correlation network containing all the m7G-related genes was presented; *EIF4E1B*, *EIF4E2*, *EIF4E*, *NCBP1*, and *NCBP2* were identified as hub genes. **Figure 2D** shows the mutations of m7G regulators based on the TCGA LUAD cohort of different datasets.

### Tumor classification and comparison based on DEGs

**Figure 3:**
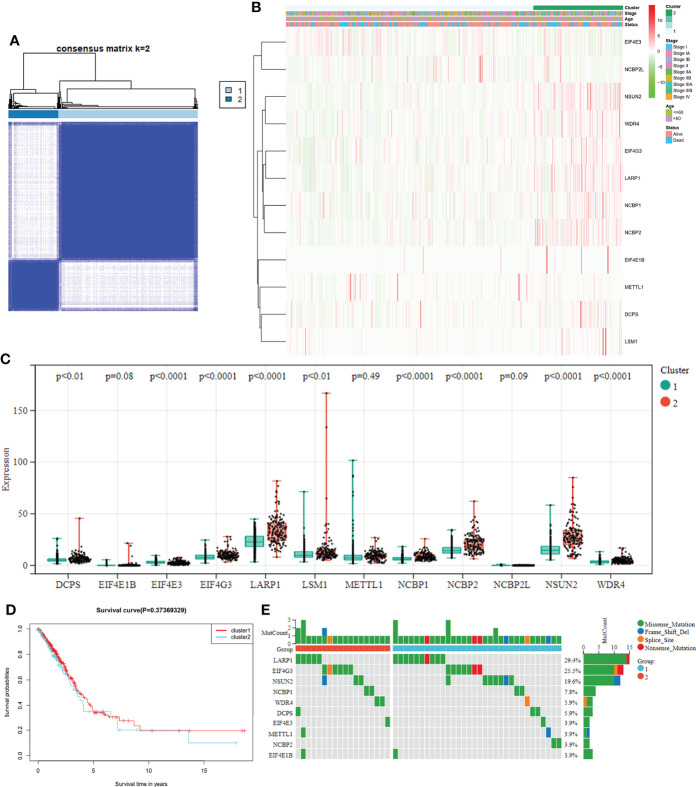
**(A)** Tumor classification based on the m7G-related DEGs. LUAD patients were grouped into two clusters according to the consensus clustering matrix (k = 2). **(B)** Heatmap and the clinicopathological characteristics of the two clusters are classified by these DEGs. **(C)** Comparisons of the expression levels of the differently expressed m7G-related genes between the two clusters. **(D)** K-M OS curves for the two clusters. **(E)** Comparisons of the mutation status in differently expressed m7G-related genes between the two clusters.

To explore the connections between the expression of the 12 m7G-related DEGs and LUAD, a consensus clustering analysis was conducted with all LUAD in the TCGA cohort. After increasing the clustering variable (k) from 2 to 10, we found that the intragroup correlations were the highest, and the intergroup correlations were lowest when the value of k = 2. The TCGA cohort of LUAD could be divided into two clusters based on 12 DEGs (**Figure 3A**). The heatmap displayed the gene expression profile and the clinical features, such as tumor stage, age (≤60 or > 60 years), and survival status (alive or dead). No significant difference was observed in the clinical features between the two clusters (**Figure 3B**). The overall survival (OS) time was also compared between the two clusters, but no obvious differences were detected (P = 0.374, **Figure 3D**). We also examined the expression of these DEGs and mutation rate between the two clusters (**Figures 3C, E**). The m7G-related genes in cluster 1 were underexpressed compared to cluster 2, while the mutation rates were reversed. Thus, whether both the expression and mutational status of these genes can affect the prognosis need further experimental and clinical investigations.

### Development of a prognostic gene model in the TCGA cohort

**Figure 4:**
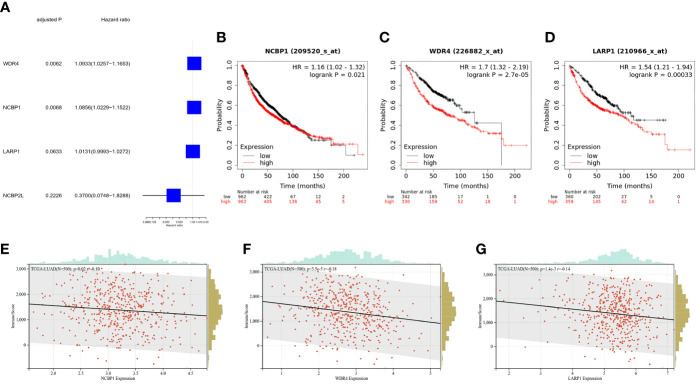
Clinical signature of the extracted m7G-related genes for risk model. **(A)** Univariate Cox regression analysis of m7G-related genes for risk model. **(B–D)** K-M survival analyses for OS among LUAD stratified by gene expression level. **(E–G)** Immune score of m7G-related genes for the risk model in LUAD, the relationships between immune score and expression were all negative.

The gene expression levels of 482 LUAD samples were submitted for primary screening of survival-related genes using univariate Cox regression analysis. To avoid omission, we set the criteria to 0.2 and included LARP1 and NCBP2L in the risk model development (**Figure 4A**). The 4-gene signature was built according to the optimum λ value employing LASSO Cox regression analysis. The risk score was calculated as follows: risk score = (0.001013 × LARP1 exp.) + (-0.715684 × NCBP2L exp.) + (0.068453 × WDR4 exp.) + (0.059285 × NCBP1 exp.). Next, we analyzed these extracted gene signatures in LUAD and found that overexpression was related to a poor survival outcome (**Figures 4B–D**) and a lower immunologic infiltration score in LUAD *via* the UCSC dataset (**Figures 4E–G**).

**Figure 5:**
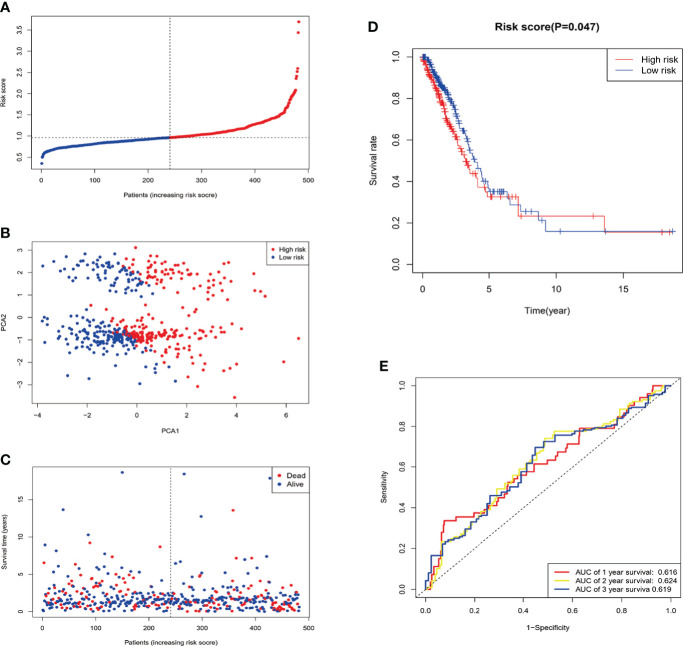
Construction of the risk signature in the TCGA cohort. After Four candidate genes obtained by LASSO regression, the risk score is computed base on these genes. **(A)** Distribution of patients based on the risk score. **(B)** PCA plot for LUAD in the entire TCGA dataset based on the risk level. **(C)** Survival status of each patient (low-risk population: on the left side of the dotted line; high-risk population: on the right side of the dotted line). **(D)** Survival analysis for OS in the low- and high-risk groups. **(E)** ROC curves demonstrated the predictive efficiency of the risk score.

Patients were divided into low- and high-risk subgroups based on the median score calculated by the risk score formula (**Figure 5A**). The clinal parameters between the two groups are summarized in **Table 1**, and no significant differences were detected in the clinical features between the two groups. PCA showed that patients with different risks were well-separated into two groups (**Figure 5B**). Patients in the high-risk group had more deaths and shorter survival time than those in the low-risk group (**Figures 5C**, **D**, P = 0.047). A time-dependent receiver operating characteristic (ROC) analysis was applied to evaluate the sensitivity and specificity of the prognostic model. Consequently, the area under the ROC curve (AUC) was 0.616 for 1-year, 0.624 for 2-year, and 0.619 for 3-year survival (**Figure 5E**), confirming the sensitivity of the risk model.

**Table 1:** Comparison of clinical parameters between the low-risk and the high-risk groups.

Covariates	Type	Total	Low-Risk	High-Risk	P-value
Age	≤60	153 (31.74%)	84 (34.85%)	69 (28.63%)	0.1707
	>60	329 (68.26%)	157 (65.15%)	172 (71.37%)	
Gender	Female	263 (54.56%)	133 (55.19%)	130 (53.94%)	0.8548
	Male	219 (45.44%)	108 (44.81%)	111 (46.06%)	
stage	Stage I	261 (54.15%)	122 (50.62%)	139 (57.68%)	0.1522
	Stage II	117 (24.27%)	57 (23.65%)	60 (24.9%)	
	Stage III	79 (16.39%)	46 (19.09%)	33 (13.69%)	
	Stage IV	25 (5.19%)	16 (6.64%)	9 (3.73%)	
T	T1	165 (34.23%)	79 (32.78%)	86 (35.68%)	0.8172
	T2	253 (52.49%)	128 (53.11%)	125 (51.87%)	
	T3	44 (9.13%)	21 (8.71%)	23 (9.54%)	
	T4	17 (3.53%)	10 (4.15%)	7 (2.9%)	
	Unknown	3 (0.62%)	3 (1.24%)	0 (0%)	
N	N0	312 (64.73%)	151 (62.66%)	161 (66.8%)	0.2146
	N1	90 (18.67%)	44 (18.26%)	46 (19.09%)	
	N2	68 (14.11%)	40 (16.6%)	28 (11.62%)	
	N3	2 (0.41%)	2 (0.83%)	0 (0%)	
	Unknown	10 (2.07%)	4 (1.66%)	6 (2.49%)	
M	M0	316 (65.56%)	160 (66.39%)	156 (64.73%)	0.363
	M1	24 (4.98%)	15 (6.22%)	9 (3.73%)	
	Unknown	142 (29.46%)	66 (27.39%)	76 (31.54%)	

### Independent prognostic value of the risk model

**Figure 6:**
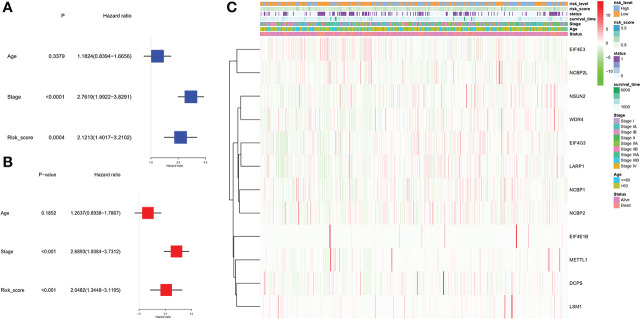
Univariate and multivariate Cox regression analyses for the risk score. **(A)** Univariate analysis for the TCGA cohort (stage: the degree of tumor stage). **(B)** Multivariate analysis for the TCGA cohort. **(C)** Heatmap (green: low expression; red: high expression) for the connections between clinicopathological features and the risk groups. We divided stages I and II as group 1 and the remaining as group 2.

Univariate and multivariable Cox regression analyses were used to evaluate whether the risk score derived from the gene signature model could serve as an independent prognostic factor. The univariate Cox regression analysis indicated that both the risk score and stage were independent significant prognostic factors predicting poor survival in the TCGA cohorts (hazard ratio (HR) = 2.1213, 95% confidence interval (CI): 1.4017-3.2102; HR = 2.7619, 95% CI:1.9922−3.8291, respectively; **Figure 6A**). And they were further proved by multivariate analysis (**Figure 6B**). Combined with the P-value of univariate analysis, age was not included in multivariate analysis. In addition, we generated a heatmap of clinical features (**Figure 6C**) and found that the age and the survival status of the patients were equivalent between the low- and high-risk subgroups (**Table 1**).

### Functional analyses based on the risk model

**Figure 7:**
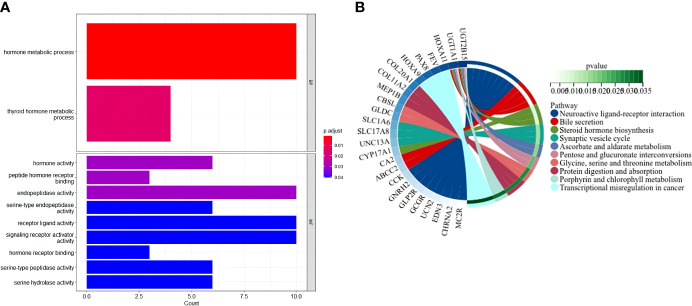
Functional analysis based on the DEGs between the two-risk groups in the TCGA cohort. **(A)** Bar graph for GO enrichment (the taller bar means more genes are enriched, and the intensity of the color means the differences were obvious; q-value is the adjusted p-value). **(B)** Circle diagram for KEGG pathways enrichment.

The “limma” R package was used to extract DEGs, and FDR< 0.05 and |log_2_FC | ≥ 1 criteria were applied to further investigate the variations in gene functions and pathways between the risk model subgroups. In the TCGA cohort, 128 DEGs were identified between the low- and high-risk groups. In the high-risk group, 83 genes were upregulated, while 45 genes were downregulated. These DEGs were then used for gene ontology (GO) enrichment analysis and Kyoto Encyclopedia of Genes and Genomes (KEGG) pathway analysis. The findings revealed that DEGs were primarily enriched in functional categories and pathways linked to hormones and metabolism (**Figure 7**).

### Comparison of the mutations and immune activity between subgroups

**Figure 8:**
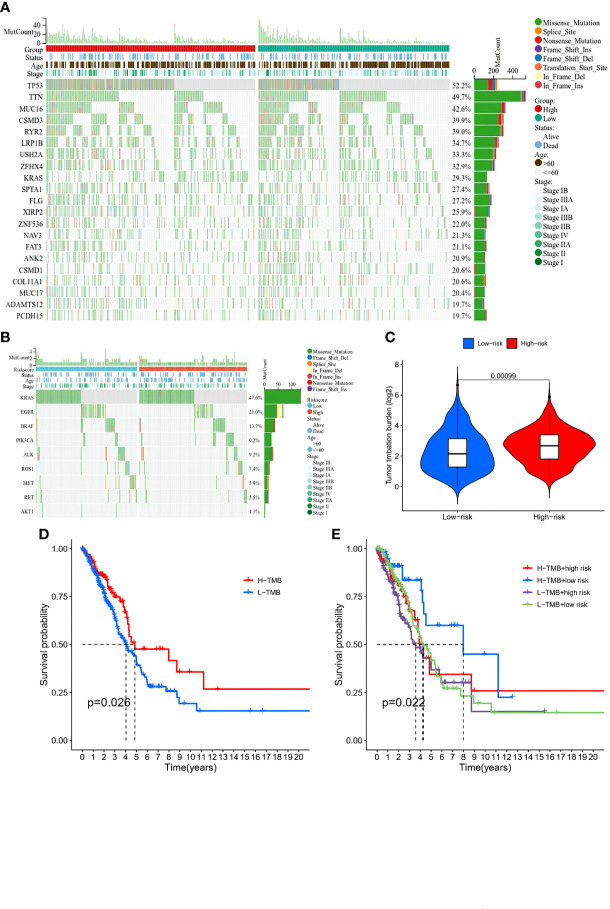
Comparison of immunity and genetic characteristics between high- and low-risk score groups based on the TCGA LUAD cohort. **(A, B)** Comparison of the mutation status between low- (green box) and high-risk (red box) groups in the TCGA cohort. **(C)** Tumor mutation burden between the two groups and patients with high risk had high burden. **(D)** Comparison of the survival probability with different levels of tumor mutational burden. **(E)** The survival probability with different levels of tumor mutational burden and risk score.

**Figure 9:**
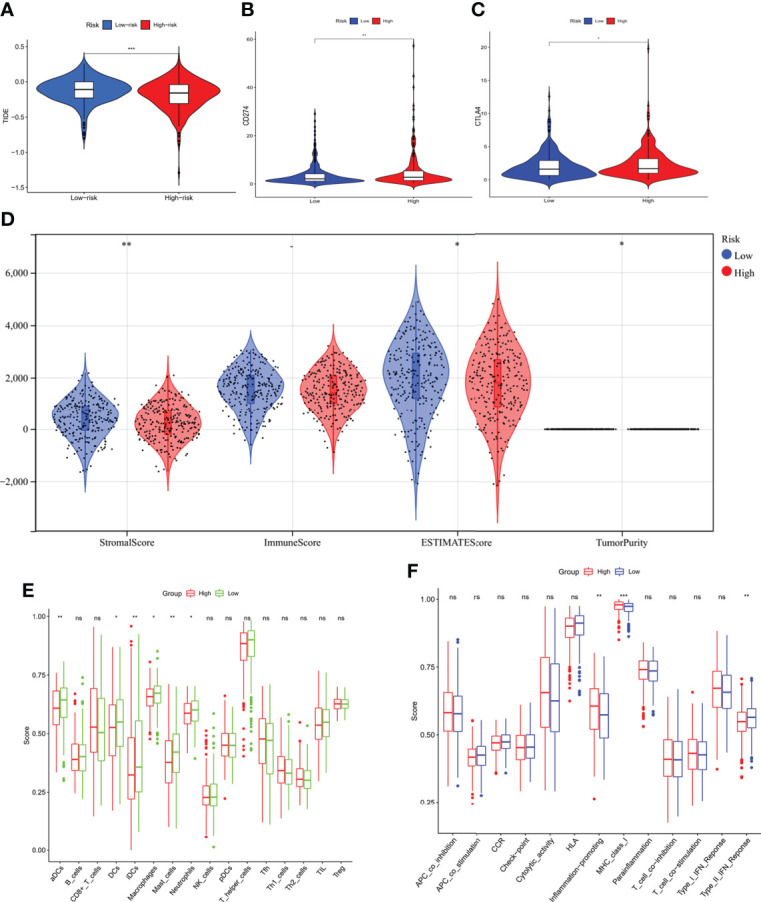
Comparison of immunity characteristics between high- and low-risk score groups based on the TCGA LUAD cohort. **(A)** comparison of the tumor immune dysfunction and exclusion between the low- and high-risk groups indicated a better immune response in the high-risk group. **(B, C)** Comparison of the immune checkpoints between low- and high-risk groups; the expression of PD-L1 and CTLA-4 was higher in the high-risk group. **(D)** Immune score comparison between the two groups to estimate the difference in the immune microenvironment. **(E, F)** Enrichment scores of 16 types of immune cells and 13 immune-related pathways between low- and high-risk groups in the TCGA. *P < 0.05; **P < 0.01; ***P < 0.001.

The comparison of the mutations between the subgroups showed a higher TMB in the high-risk group than the low-risk group, as well as some oncogenes, including *KRAS*; also, the survival probability in high TMB was better, especially for those with a low-risk level (**Figure 8**). Reportedly, m7G could reshape the microenvironment, especially the immune cell infiltration (15). Combined with functional analyses, we further compared the enrichment scores of 16 types of immune cells and the activity of 13 immune-related pathways between the low and high-risk groups employing ssGSEA. In the TCGA cohort (**Figures 9E, F**), the high-risk subgroup had lower infiltration of immune cells, including dendritic cells (DCs), induced DCs (iDCs), neutrophils, and macrophages, than the low-risk subgroup. Regarding the immune-related pathways, the scores of inflammation-promoting and MHC-class I cells were higher in the high-risk group, while the type II interferon (IFN) response was lower (**Figures 9E, F**). Compared to the low-risk group, the mutation state of oncogenes, including *EGFR* and *MET*, was significantly different in the high-risk group, indicating their potential role in predicting the efficacy of target therapy. In addition to the well-known predictors for ICIs, newly identified predictors, such as TIDE, are frequently employed and strongly advised for evaluating the immune response and immune evasion. Also, the expression of PD-L1 and CTLA4 was higher in the high-than the low-risk group (**Figures 9A–C**). Furthermore, we compared the degree of stromal cell infiltration (stromal score) across three unique patterns. As an immune desert, high-risk patients had higher stromal scores compared to low-risk patients, indicating that high-risk LUAD had more nontumor components, such as immune cells and stromal cells, indicating a higher tumor purity (**Figure 9D**).

### Prediction value in anticancer therapy

**Figure 10:**
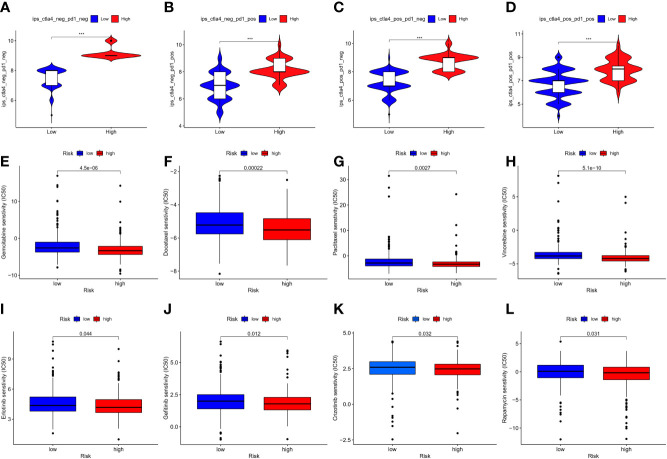
**(A–D)** represented immunophenoscore of LUAD to predict response to anti-cytotoxic T lymphocyte antigen-4 (CTLA-4) and anti-programmed cell death protein 1 (anti-PD-1) antibodies, and the high-risk group seemed to be better in immunotherapy; **(E–L)** indicated the comparison of the drug sensitivity between low- and high-risk score groups, the lower the IC50, the more the sensitivity. *P < 0.05; **P < 0.01; *** P < 0.001.

In the current study, TIDE was significantly elevated in the low-risk group, indicating that immunotherapy was less effective (16), which was consistent with the immunophenoscore (IPS) analyses (**Figures 10A–D**). Owing to the shortage of PD-L1 in predicting the efficacy of immunotherapy, whether our model could be better in prediction is to be explored. The results demonstrated a crucial role of m7G in mediating the clinical response to ICI treatment by the impact on TMB, immune cell infiltration, immunogenicity, and checkpoint expressions. These features might provide insights into the m7G-regulated immune microenvironment in LUAD and identify numerous potential immunotherapeutic targets. Regarding common drug sensitivity, including the chemotherapy and target therapy, we found that high-risk group was significantly more sensitive to Gemcitabine, Docetaxel, Paclitaxel, Crizotinib, Erlotinib, Gefitinib, and Rapamycin than the low-risk group (**Figures 10E–L**). Moreover, Rapamycin is an mTOR inhibitor, which was in agreement with a previous study, wherein METTL1 accelerated proliferation and autophagy through the AKT/mTORC1 signaling cascade (17).

## Discussion

m7G is a methyl group added to the seventh N of RNA guanine, increasing the RNA stability (18). The dysregulation of tRNA underlies cancer development and is associated with a high metabolic and proliferative status, resulting in dysregulation of biological and pathological functions (19). Currently, the underlying mechanisms of m7G modification in cancer are not understood comprehensively; thus, we investigated the potential value of the m7G-related genes in diagnostic and therapeutic strategies for LUAD.

Herein, the mRNA expression of these 29 m7G-related genes in control and cancer samples was elevated. The two groups formed by the consensus clustering analysis of DEGs did not exhibit any statistically significant differences in survival time. In order to elucidate the function of these DEGs, we used Cox univariate and LASSO Cox regression analysis to develop a four-gene risk model. Based on the model’s score, the data were divided into low- and high-risk groups. The survival rates were better in the low- than the high-risk group. In both univariate and multivariate studies, the risk score was determined as an independent factor, and the ROC curve indicated its sensitivity. Functional investigations indicated that the DEGs between the two subgroups were associated with metabolic pathways, and some of the DEGs were implicated in cancer transcriptional dysregulation. We also examined the genetic features of high- and low-risk individuals and found that the high-risk group had a greater rate of somatic mutations in multiple genes, including *TP53*, *KRAS*, and *MET*.

Tumorigenesis is the process wherein a tumor begins and grows outside the limits of an organ or tissue. The effects of RNA on writers, readers, and erasers may contribute to or avoid certain cancer traits. Accumulating evidence shows that RNA changes and the enzymes involved in their deposition, clearance, and detection, play diverse roles in various malignancies (20). In a recent study, METTL1 or WDR4 knockdown in mouse embryonic stem cells resulted in a poor self-renewal capacity and a disrupted differentiation program, demonstrating its physiological role in mammalian systems. (12). In addition to physiology, m7G plays a critical role in cancer. Also, m7G methyltransferase WD repeat domain 4 (WDR4) expression was abnormal in various malignancies and was linked to OS and immune infiltration, according to a pan-cancer investigation (21). Other studies demonstrated that another component of the tRNA m7G methyltransferase complex, methyltransferase-like 1 (METTL1), was upregulated in some malignancies, such as hepatocellular carcinoma and lung adenocarcinoma, and was associated with poor patient prognosis and resistance to chemotherapy (22, 23). Another study showed high METTL1 and WDR4 expression levels in lung cancer, facilitating m7G tRNA modification, altering mRNA translation, and boosting lung cancer development and invasion (24). In the current study, we verified that the DEGs, *METTL1*, and *WDR4*, were upregulated in the TCGA cohort. Moreover, WDR4 was extracted for risk model construction. Its high expression was related to poor outcomes in LUAD across the K-M survival curve, indicating its role in cancer, especially lung adenocarcinoma. In the current analysis, eIF4E3 was downregulated in LUAD compared to the normal samples. A model indicated that eIF4E3 acted as a tissue-specific tumor suppressor, repressing oncogenic transformation, and cancer could be driven by the loss of the suppressive activity of eIF4E3 (25). Another gene, the La-related protein 1 (*LARP1*), has been shown to interact with 3000 mRNAs linked to cancer pathways, including post-transcriptionally controlled mTOR which was frequently dysregulated in cancer, promoting cell motility, invasion, and anchorage-independent growth. (26). Furthermore, interaction with the 3’-untranslated regions (3’-UTRs) stabilized BCL2, encouraging ovarian cancer growth and chemotherapy resistance (27). In lung adenocarcinoma, we found that high expression levels of LARP1 are correlated with poor survival and nuclear cap-binding protein 1 (NCBP1). Interestingly, NCBP1 is required for capped RNA synthesis and intracellular translation, and has recently been discovered to interact with NCBP3 to induce CUL4B expression, promote lung cancer cell growth, wound healing, migration, and epithelial-mesenchymal transition (28). Although several studies discovered the link between the sophisticated molecular roles of tRNA alterations, selective mRNA translational, control, and human cancer, a few underlying molecular pathways are functionally related to specific tRNAs and the network changes in human cancer (11). In the present study, we evaluated the role of the m7G regulator in lung adenocarcinoma. The K-M and ROC curves demonstrated that the risk model based on these regulators performed adequately, although an in-depth analysis is required.

The functional analysis of the DEGs between the risk subgroups in the TCGA cohort revealed that some genes were enriched in the hormone and metabolism-related functional categories and pathways, implying that they may regulate some hormone-related cancers, such as prostate cancer. Prostate cancer development, growth, and metastasis depend initially on androgens. The study indicated that two major pathways involved in prostate cancer progression, PI3K/Akt/mTOR, and Ras/MAPK, intersect at the eukaryotic transcription initiation factor eIF4E. Furthermore, phosphorylation of eIF4E increased the rate of translation of oncogenic mRNAs, increasing tumorigenicity and promoting resistance to chemotherapy and endocrine therapy (29). While METTL1 also shared this mechanism, it boosted A549 cell growth and colony formation by inhibiting autophagy *via* the Akt/mTOR pathway (23). In addition to hormone-related tumors, several factors, including inactivating mutations in tumor suppressors (TP53) and activation of oncogenes (*EGFR* or *MYC*) in this study, provided clinical insights into m7G in lung cancer. A recent study showed that WDR4 and WDR4-related m7G methylation levels were upregulated in addition to the common mechanisms of epithelial-mesenchymal transition, activation of G2/M cell cycle transition, and apoptosis inhibition. Another study showed that MYC triggered WDR4 transcription, thereby stabilizing and initiating the translation of *CCNB1* mRNA, which in turn increased PI3K and AKT phosphorylation and decreased P53 protein levels (30). In the high-risk group, a high mutation rate of KRAS and MYC was detected, which could be an orientation for further mechanism and treatment-related studies or extract patient benefits from target therapy.

Several physiological and pathological processes, including the maturation of immune cells and immune response, are influenced by RNA methylation (18). As stated previously, m7G-related genes are associated with immune infiltration, while the current findings indicated that the low-risk group has a high level of immune cell infiltration, especially in different types of DCs that are antigen-presenting cells with critical roles in the initiation and regulation of both innate and adaptive immune responses. DCs also improve immunization and tolerance by presenting antigens to T cells and sending immunomodulatory signals *via* cytokines (31). In the TME, before forming T cell responses, DCs needed to receive, process, and display tumor-associated antigens on MHC molecules and offer co-stimulation and soluble factors (32). Another study showed that RNAs with methylation modifications inhibited DC activation, and the higher the level of modification, the fewer cytokines and activation factors. This alteration suppressed the potential of RNA to activate DCs (33). Next, we extracted the single risk model-related genes for immune score analyses; the high gene expression was correlated with a low score. Additionally, most immune-related pathways did not exhibit significant differences except for the inflammation promotion, MHC class I, and type II IFN response. JAK-STAT signaling pathway was activated by type II IFN and exerted critical roles in both innate and adaptive immunity (34). Moreover, type II IFN was not involved in the development of cancer immunotherapy treatments due to its ability to prevent tumor growth (35). Also, the immune scores were significantly higher in the low-risk group than the high-risk group and reflected a better outcome, while the high-risk group indicated immune escape.

Many studies have focused on the prevention and early detection of cancer and anticancer therapy. However, the intricate mechanism restricts therapeutic efficiency. Also, RNA misregulation may play a role in the cancer process, including anticancer drug resistance. Typically, tRNA overexpression in malignancies can block apoptosis by binding to cytochrome-c and limiting caspase activation, resulting in castration-resistant prostate cancer (CRPC) (19). The eukaryotic translation initiation factor eIF4E is increased in 30% of cancers, including the M4/M5 subtypes of acute myeloid leukemia. Furthermore, in leukemic blasts, a therapeutic study targeting eIF4E exhibited clinical efficacy and related molecular responses, but only 2/11 patients had disease progression (36). Thus, we questioned whether finding some biomarkers to enhance efficiency or a biomarker for predicting immunotherapy efficacy would be beneficial. In this study, we assessed the correlations between PD-L1 and CTLA4 with m7G regulators and found that the expression levels were higher in the high-risk group. As described previously, m7G influences the immune cells and immune responses, and those who tolerated immunotherapy had more methyltransferases in lung cancer treatment (37). The immune checkpoint and TIDE indicated that the high-risk group could benefit from immunotherapy. In addition, the drug sensitivity analyses might guide drug therapy. Taken together, the current findings suggested that m7G regulators or the risk model could be employed as a prognosis assessor as well as a biomarker for LUAD patients who would benefit from anticancer therapy, although additional investigations are essential.

## Conclusions

In conclusion, this study demonstrated the importance of m7G modification in LUAD and emphasized the vital role of m7G modification in shaping the heterogeneity and complexity of the tumor microenvironment. Furthermore, the risk signature score based on four m7G-related genes constituted an independent risk factor for predicting OS. Notably, the current findings have created a new gene signature for predicting the prognosis of LUAD patients. They also orient new studies into the links between m7G-related genes and the LUAD microenvironment, improving the understanding of the mechanism and drug discovery.

## Data availability statement

Publicly available datasets were analyzed in this study. This data can be found here: https://portal.gdc.cancer.gov/projects/TCGA-LUAD.

## Author contributions

YD performed most of the experiments and data analysis and drafted the manuscript. YL did the rest work like image processing. YY provided professional advice on the experiment design and reviewed the paper. QS managed the experimental design, reviewed the manuscript, and provided funding support. All authors contributed to the article and approved the submitted version.

## Funding

Beijing Medical and Health Foundation (No. YWJKJJHKYJJ-BXS5-22006); Wu Jieping Medical Foundation (No. 320.6750.19094-18); Beijing Health Alliance Charitable Foundation (No. YXKY-WS834B); Key Youth Training Foundation of Renmin Hospital of Wuhan University (No. 2013-18).

## Acknowledgments

We would like to thank all the authors for their help.

## Conflict of interest

The authors declare that the research was conducted in the absence of any commercial or financial relationships that could be construed as a potential conflict of interest.

## Publisher’s note

All claims expressed in this article are solely those of the authors and do not necessarily represent those of their affiliated organizations, or those of the publisher, the editors and the reviewers. Any product that may be evaluated in this article, or claim that may be made by its manufacturer, is not guaranteed or endorsed by the publisher.
